# Combined Dietary Effects of *Tenebrio molitor* Meal and Probiotics on Growth Performance, Immune Response, and Pathogen Resistance in Nile Tilapia (*Oreochromis niloticus*)

**DOI:** 10.1155/anu/5390688

**Published:** 2026-08-09

**Authors:** Sivaramasamy Elayaraja, Vlastimil Stejskal, Raja Subramaniyan, Santosh Kumar Karn, Mahmoud Mabrok, Channarong Rodkhum

**Affiliations:** ^1^ Center of Excellence in Fish Infectious Diseases (CE FID), Faculty of Veterinary Science, Chulalongkorn University, Bangkok 10330, Thailand, chula.ac.th; ^2^ University of South Bohemia in Ceske Budejovice, Faculty of Fisheries and Protection of Waters, South Bohemian Research Center of Aquaculture and Biodiversity of Hydrocenoses, Zátiší 728/II, Vodňany 389 25, Czech Republic, jcu.cz; ^3^ Atal Centre for Ocean Science and Technology for Islands, National Institute of Ocean Technology, Sri Vijaya Puram (Port Blair) 744 103, Andaman and Nicobar Islands, India, niot.res.in; ^4^ Department of Biochemistry and Biotechnology, Sardar Bhagwan Singh University, Dehradun 248001, India; ^5^ Department of Aquatic Animal Medicine, Faculty of Veterinary Medicine, Suez Canal University, Ismailia 41522, Egypt, scuegypt.edu.eg

**Keywords:** disease resistance, growth performance, Nile tilapia, probiotics, *Tenebrio molitor*

## Abstract

The prospective utilization of insects as alternative protein sources to replace fishmeal (FM) in aquafeed has prompted increased interest. This study evaluated the effects of yellow mealworm *Tenebrio molitor* (TML)‐based diet supplemented with 2% mixed probiotics on growth performance and resistance to *Aeromonas hydrophila* infection in Nile tilapia (*Oreochromis niloticus*). Six experimental diets were formulated. The control group (CTRL) received commercial feed containing 30% FM. In the experimental groups (T25P^+^, T50P^+^, and T100P^+^), FM was replaced with TML at inclusion rates of 25%, 50%, and 100%, respectively. The T50P^–^ group was identical to the T50P^+^ group but lacked probiotics, whereas the CTRLP^+^ group received the control diet (without TML) supplemented with 2% probiotics. Juvenile tilapia weighing 12.81 ± 1.37 g were fed the respective diets for 8 weeks. By the end of the feeding trial, fish receiving the T50P^+^ diet exhibited superior overall performance, characterized by a significantly higher weight gain (WG; 91%) and survival rate (98%) compared with the CTRL (*p* < 0.05). This group also demonstrated markedly enhanced digestive enzyme activities, including protease (30.5–34.7 U/mg), lipase (18.2–22.6 U/mg), and amylase (20.8–23.5 U/mg), alongside elevated lysozyme activity, improved antioxidant capacity, and stronger bactericidal activity. Although the T100P^+^ diet led to improvements in several parameters relative to the control, its overall performance remained inferior to that of the T50P^+^ group, indicating that partial replacement of FM was more effective than complete substitution. Furthermore, fish fed the T50P^+^ diet displayed enhanced fillet quality, evidenced by higher crude protein (CP) content (59.63%) and a moderate lipid level (29.73%). Following challenge with *Aeromonas hydrophila*, the T50P^+^ group showed a substantially reduced mortality rate (20%) compared with the CTRL (80%), confirming a pronounced improvement in disease resistance. Overall, the results suggest that replacing 50% of FM with TML in combination with probiotics enhances growth, nutrient utilization, immune function, and pathogen resistance in Nile tilapia. This TML–probiotic formulation represents a promising, sustainable, and effective alternative to conventional aquafeeds.

## 1. Introduction

Nile tilapia (*Oreochromis niloticus*) is one of the most commercially vital species in global aquaculture, where the annual production exceeds 6 million tons and is worth more than 9 billion USD to the global economy [1, 2]. Its rapid growth, ecological adaptability, and high nutritional value have established it as a crucial species in both small‐scale and industrial aquaculture. Nevertheless, Nile tilapia are vulnerable to bacterial infections, particularly *Aeromonas hydrophila*, which may cause extensive production losses, low feed efficiency, and considerable mortality rates [3]. This pathogen, which causes hemorrhagic septicemia, tissue necrosis, and liver damage, presents a significant threat to the global sustainability of tilapia farming [4]. Frequent outbreaks of *A. hydrophila* have prompted the widespread application of antibiotics in aquaculture; however, this has raised serious concerns about the emergence of antimicrobial resistance and associated environmental risks [5, 6].

Multidrug resistance (MDR) has increased globally and now represents a critical public health challenge. Indeed, in recent years, multidrug‐resistant bacteria from different origins have been increasingly reported, highlighting the importance of the prudent use of antibiotics [7, 8]. These consequences undermine the effectiveness of disease control efforts and threaten the safety of our food and public health. With increasing concerns over the misuse of antibiotics, there is a pressing need to adopt sustainable and eco‐friendly approaches to enhance fish growth, boost immunity, and prevent disease outbreaks. Recently, attention has turned to nonantibiotic alternatives. Approaches such as phage therapy, vaccines, gene editing, immunostimulants, and the addition of herbal medicines to fish diets show promise in improving overall fish health and nutrition [9–11].

The incorporation of bioactive components, such as insect‐based proteins and probiotics, into fish feed has emerged as a promising strategy. These ingredients have been demonstrated to promote growth, enhance immune function, and improve disease resistance to a variety of diseases [12, 13]. Yellow mealworms (*Tenebrio molitor* larvae [TML]) have gained attention as highly promising alternative protein sources. They boast a rich nutritional profile, with protein contents ranging from 50% to 60%, along with essential amino acids (AAs), valuable fatty acids (FAs), and antimicrobial peptides such as tenecin, and the polysaccharide chitin [14, 15]. Indeed, adding insect protein to fish diets is becoming widely recognized for its potential to improve gut health, increase nutrient absorption, and regulate the fish immune response [16, 17].

Similarly, probiotics are commonly used because of their ability to improve the immune function and digestive health. They have consistently been shown to be effective in promoting the growth of beneficial gut microbiota, suppressing harmful bacteria, and regulating fish immune responses, which in turn enhances disease resistance and overall performance in aquaculture [18]. Among the diverse probiotic candidates, *Bacillus subtilis*, *Lactobacillus* spp., and *Enterococcus gallinarum* have been extensively applied in aquaculture due to their demonstrated ability to modulate the gut microbiota, enhance host immune responses, and improve resistance against bacterial pathogens [19]. Supplementing these probiotics with fish diets has been linked to better growth performance and higher survival rates when fish are exposed to disease challenges [20].

Insect‐derived proteins such as TML have recently emerged as promising alternative feed ingredients owing to their high protein content and potential functional attributes [20]. Beyond their nutritional value, insect meals are rich in bioactive compounds that may enhance immune function and promote gut health [21, 22]. Despite these advantages, their combined application with probiotics in tilapia nutrition remains inadequately investigated. Although both insect‐based meals and probiotics have been extensively studied independently in aquaculture, there is a notable lack of information regarding their combined effects in Nile tilapia diets. In particular, it remains unclear whether the concurrent inclusion of TML and probiotics can synergistically improve the growth performance, digestive efficiency, and resistance to bacterial pathogens such as *Aeromonas hydrophila*. Accordingly, the present study assessed graded levels of fishmeal (FM) replacement with TML, with and without probiotic supplementation, to determine the optimal dietary strategy. This approach is intended to enhance aquaculture productivity while supporting the development of more resilient and sustainable farming systems.

## 2. Materials and Methods

### 2.1. Experimental Fish and Husbandry

A total of 500 healthy *O. niloticus* (average weight of 12.81 ± 1.37 g), with no known history of infection, were obtained from a commercial tilapia hatchery in Thailand and transported in oxygenated plastic bags to the experimental aquaculture facility at Chulalongkorn University (CE FID). Upon arrival, the fish were stocked in 1000 L fiber tanks and acclimated for 2 weeks under controlled conditions. The water temperature was maintained at 28°C, with a pH of 7.0–7.5 and dissolved oxygen (DO) levels between 6 and 7 mg/L, supported by continuous aeration. During acclimation, the fish were fed a commercial diet containing 32% crude protein (CP) to meet basic nutritional needs.

### 2.2. Collection, Drying, and Defatting of TML

TML were sourced from a local market in Chatuchak, Bangkok. The drying and defatting procedures were adapted from [22, 23], with adjustments made to suit the objectives of this study. Larvae were first thoroughly rinsed with double‐distilled water (DDW) to eliminate surface contaminants and then chopped into smaller segments to facilitate fat removal. Mechanical pressing was applied to extract lipids, thereby increasing the CP concentration and enhancing the nutritional value of the final product. The defatted samples were immediately frozen, followed by oven‐drying at 60 °C for 24 h to improve palatability. The dried larvae were subsequently ground into a fine powder via a laboratory mill and stored in sealed polyethylene bags at freezing temperatures until being incorporated into the experimental diets.

### 2.3. Bacterial Culture and Preparation

*Bacillus subtilis* was isolated from the gut of healthy Nile tilapia. Briefly, gut tissues were aseptically excised, homogenized, and serially diluted in sterile phosphate‐buffered saline (PBS). Aliquots were then plated on nutrient agar and incubated aerobically at 37 °C for 48 h to promote *Bacillus subtilis* (CEFID‐P3) growth. *Enterococcus gallinarum* (WS2021) was obtained from Cintex Lab, Bangkok. *Aeromonas hydrophila*, originally isolated from infected fish, *was kindly provided by the Fish Infectious Research Unit at Chulalongkorn University*. The *A. hydrophila strain* (CUVET46) was confirmed through Gram staining, conventional biochemical profiling for pathogen specificity, MALDI‐TOF mass spectrometry, and 16S rRNA gene sequencing. All bacterial isolates were initially cultured on tryptic soy agar (TSA) at 28 °C for 24 h. Pure cultures were preserved in tryptic soy broth with 20% glycerol at –80 °C for long‐term storage. For dietary supplementation, *B. subtilis* and *E. gallinarum* were incorporated into the feed at a minimum concentration of ≥1 × 10^8^ CFU/g feed, while *A. hydrophila* was used in bactericidal activity assays.

### 2.4. Experimental Diet Preparation

The experimental diets were formulated following the nutritional frameworks and methodologies described by Stejskal et al. and Naveed et al. [16, 24]. The probiotic blend consisted of *B. subtilis* (isolated from the tilapia gut) and *Enterococcus gallinarum* (Cintex Lab) and was administered at ≥1 × 10^8^ CFU per gram of feed. The 2% inclusion level was selected on the basis of previous studies demonstrating optimal benefits for tilapia growth and immune function [25, 26]. Six diets were created with varying TML levels and probiotics (Table 1). The control group (CTRL) received commercial feed containing 30% FM without TML or probiotics. The T25P+, T50P+, and T100P+ diets contained 2% probiotics, replacing 25%, 50%, and 100% of FM with TML meal, corresponding to dietary inclusion levels of 7.5%, 15%, and 30%, respectively. The T50P^–^ diet contained the same TML level (15%) but without probiotic supplementation, whereas the CTRLP^+^ diet consisted of a commercial feed with 2% probiotics but no TML (Table 2). The experimental diets were processed via a single‐screw extruder to produce floating pellets, which were subsequently oven‐dried at 40 °C for 12 h. The dried feeds were then stored in airtight polyethylene bags at –20 °C, following the protocol described by Sulaiman et al. [27]. The approximate composition, including the CP, lipid, ash, fiber, and moisture contents, was analyzed according to standard AOAC methods [28] and is presented in Table 2. FA profiles were determined via the lipid extraction and fatty acid methyl ester (FAME) preparation method described by Folch et al. [29], which employs methanol containing 2% sulfuric acid as the methylating agent.

**Table 1:** Experimental design and groups.

Experimental setup	Diet details	TML inclusion levels (%)
CTRL	Standard commercial feed containing 30% FM with no TML or probiotics	—
T25P^+^	25% FM with TML with 2% probiotics	7.5
T50P^+^	50% FM with TML with 2% probiotics	15
T100P^+^	100% FM with TML with 2% probiotics	30
T50P^−^	50% FM with TML without any probiotics	15
CTRLP^+^	Control diet plus 2% probiotics without TML.	—

*Note:* P^+^ = probiotics involved; P^-^ = without probiotics.

Abbreviations: CTRL, control; TML, *Tenebrio molitor* larvae.

**Table 2:** The formulation and proximate composition (%) of the experimental diets contained different levels of TML and probiotics.

Ingredients (%)	CTRL	T25P^+^	T50P^+^	T100P^+^	T50P^–^	CTRLP^+^
Corn gluten meal (CGL)	8.0	8.0	9.0	6.0	9.0	7.0
Fish oil (FO)	1.0	1.0	1.0	1.0	1.0	2.0
Fishmeal (FM)	30.0	22.5	15.0	0.0	15.0	30.0
TML	0.0	7.5	15.0	30.0	15.0	0.0
Merigel	6.0	6.0	6.0	6.0	6.0	6.0
Promix (probiotics)	0.0	2.0	2.0	2.0	0.0	2.0
Soybean meal (SBM)	18.0	18.0	18.0	19.0	18.0	18.5
Soybean oil (SO)	1.0	1.0	1.0	1.0	1.0	2.0
Vitamin and mineral premix	2.5	2.5	2.5	2.5	2.5	2.5
Pigment	0.1	0.1	0.1	0.1	0.1	0.1
Wheat flour (WF)	31.9	29.9	28.4	29.9	30.4	28.4
Amino acids (AA)	1.5	1.5	2.0	2.5	2.0	1.5
Proximate composition (% on dry‐matter basis)						
Crude protein (CP)	37.6	36.4	36.2	33.3	36.2	36.9
Crude lipid (CL)	5.4	6.7	8.0	10.5	7.9	7.4
Ash	5.7	5.3	4.9	3.9	4.8	5.7
Fiber	2.4	2.6	2.9	3.6	2.9	2.3

*Note:* CTRL, control diet (with no insect meal (*Tenebrio molitor*) or probiotics); T25P^+^, T50P^+^, T100P^+^ includes 2% probiotics and substitutes 25%, 50%, or 100% FM with TML; T50P^−^, inclusion of 50% TML without probiotics; CTRLP^+^, commercial feed with 2% probiotics but no TML. The values are expressed as percentages of the diet (as‐fed basis for ingredients; dry matter basis for proximate composition).

### 2.5. Experimental Design

After the acclimatization period, uniformly sized Nile tilapia (12.81 ± 1.37 g) were randomly allocated into 18 glass tanks (250 L each) at a stocking density of 20 fish per tank. Each tank was equipped with a continuous aeration system and securely covered to prevent fish escape. The six experimental diets were randomly assigned to tanks in triplicate. The fish were fed to apparent satiation three times via the hand‐feeding method daily at 09:00, 13:00, and 17:00 h. The feeding trial was conducted over a period of 8 weeks. Water quality parameters, including DO, pH, and temperature, were monitored regularly and maintained within optimal ranges in accordance with standard guidelines [30]. Ammonia and nitrite concentrations were measured twice weekly via commercial water testing kits and were consistently maintained below 0.02 and 0.2 mg/L, respectively. To prevent water quality deterioration, the uneaten feed was removed after each feeding session. The collected feed residues were dried and weighed to accurately quantify the actual feed intake. The fish were monitored daily for signs of stress or disease, and all mortalities were recorded to calculate survival rates over the experimental period. Dead fish were not replaced during the trial.

### 2.6. Growth Performance

Fish growth performance parameters, including weight gain (WG), specific growth rate (SGR), feed conversion ratio (FCR), and survival rate, were assessed biweekly following the methodology described by Elayaraja et al. [31], with the following formulas:

(1) Weight gain WG=Final weight g−Initial weight g,

(2) Feed conversion ratio (FCR) was calculated as feed intake (g)/weight gain (g),

(3) SGR%/day=lnfinalweight−lninitialweight/days×100,

(4) Survival rate %=Final number of fish/Initial number of fish×100.

### 2.7. Sampling Procedures

At the end of the feeding trial, five fish were randomly sampled from each tank (three tanks per treatment), yielding 15 biological replicates per treatment group for blood analysis. Prior to collection, the fish were anesthetized with 100 mg/L MS‐222 (tricaine methane sulfonate, Finquel; Argent Chemical Laboratories) and buffered with 50 mg/L sodium bicarbonate to neutralizethe acidity. Blood was drawn from the caudal vein via heparinized syringes to prevent coagulation. A portion of the fresh blood was immediately utilized for hematological analysis following the methodology described by Guardiola et al. [32]. The remaining blood was allowed to coagulate and then centrifuged at 3000×*g* for 15 min to obtain the serum. The serum was carefully collected via a sterile pipette and stored at −20°C for subsequent analysis.

#### 2.7.1. Hematological Profile Analysis

Hematological parameters, including hemoglobin (Hb), hematocrit (HCT), red blood cell (RBC) count, white blood cell (WBC) count, mean corpuscular volume (MCV), and mean corpuscular hemoglobin (MCH), were analyzed in triplicate. Measurements were conducted according to the standard methods of Blaxhall et al. [33], with slight modifications.

Total RBC and WBC counts were determined via a Neubauer hemocytometer under a light microscope (Olympus CX23) at 400× magnification. RBC and WBC values were expressed as ×10^6^ cells/µL and ×10^3^ cells/µL, respectively. The hemoglobin concentration was quantified via a commercially available assay kit (Sigma‒Aldrich Heme Assay Kit, MAK316), whereas the HCT was assessed via heparinized capillary tubes and expressed as a percentage of the packed cell volume, following the procedure described by Guardiola et al. [32].

The MCV and MCH were calculated according to the formulas described by Bain et al. [34]. The calculations were as follows:

(5) MCVfL=HematocritHCT%/RBC countmillions/mm3×10

(6) MCHpg=HemoglobinHbg/dL/RBCcountmillions/mm3×10

The results are expressed in femtoliters (fL) for MCV and picograms (pg) for MCH.

#### 2.7.2. Lysozyme Activity Assay

Serum lysozyme activity was determined in triplicate via the turbidimetric assay described by Guardiola et al. [32], with slight modifications. A suspension of *Micrococcus lysodeikticus* (0.2 mg/mL) prepared in 0.05 M phosphate buffer (pH 6.2) served as the substrate. Twenty microliters (20 µL) of each serum sample was added to 2 mL of the bacterial suspension, and the decrease in turbidity was measured spectrophotometrically at 450 nm after 0 and 15 min of incubation at 25 °C. Measurements were performed using a microplate reader (Thermo Fisher Scientific, Darmstadt, Germany). Lysozyme activity was quantified by comparing the reduction in optical density (OD) with a standard curve generated using hen egg‐white lysozyme (Sigma‒Aldrich, USA) at concentrations ranging from 0 to 20 µg/mL. The results are expressed as units of lysozyme per milliliter of serum (unit/mL).

#### 2.7.3. Digestive Enzyme Assay

Digestive enzyme activities were evaluated on the basis of the procedure described by Naveed et al. [24]. Intestinal tissues were carefully excised from freshly dissected fish from each dietary group and homogenized in an ice‐cold sucrose solution. The homogenates were centrifuged, and the resulting supernatants were stored at −20 °C until the analysis. The enzyme activities of amylase, protease, and lipase were measured via a UV‒visible spectrophotometer (Hitachi U‐2001). Amylase activity was determined following the method of Rick et al. [35], in which a starch mixture was incubated with the enzyme extract at 37 °C for 30 min, and the resulting absorbance was measured at 540 nm. Protease activity was analyzed via the [36] method by incubating casein with the enzyme extract at 37 °C and measuring the absorbance of the hydrolyzed products at 280 nm. Lipase activity was measured via the method of Mahadik et al. [37], with *p*‐nitrophenyl palmitate used as the substrate, and the absorbance was recorded at 410 nm. All enzyme assays were conducted in triplicate to ensure accuracy and reproducibility. The enzyme activities were normalized to the soluble protein content of each extract, which was quantified prior to analysis and are expressed as units per milligram of protein (U/mg).

#### 2.7.4. Antioxidant Status

The activities of antioxidant enzymes, including catalase (CAT), glutathione peroxidase (GSH), superoxide dismutase (SOD), and the lipid peroxidation marker malondialdehyde (MDA), were assessed in liver samples from each replicate fish following the methods described by Ashry et al. [38]. Briefly, liver tissues were carefully dissected and homogenized in cold phosphate buffer (pH 6.5) at a tissue‐to‐buffer ratio of 1:4 (w/v). The homogenates were centrifuged at 11,200×*g* for 10 min at 4 °C, and the resulting supernatants were collected and stored at −80 °C until analysis. Antioxidant enzyme activities were quantified via colorimetric assays, with absorbance readings measured at 560 nm via a microplate reader.

#### 2.7.5. Bactericidal Activity (MTT Assay)

The bactericidal activity was determined via a modified 3‐(4,5‐dimethylthi‐azol‐2‐yl)‐2,5‐diphenyltetrazolium (MTT) assay, as described previously [9]. In brief, 100 µL of the collected serum was incubated with 100 µL of a bacterial suspension of *A. hydrophila* (10^6^ CFU/mL) at 37°C for 1 h. Following the incubation period, 10 µL of MTT (5 mg/mL) was added to the mixture and incubated for another 4 h. The reduction of MTT by viable bacteria into blue formazan was measured by its absorbance at 570 nm via a microplate reader. The intensity of the color produced is proportional to the number of surviving bacterial cells. The bactericidal activity was calculated as the percentage reduction in absorbance compared with that of the control lacking serum.

#### 2.7.6. Challenge Trial

For the bacterial challenge test, 10 fish were randomly selected from each dietary treatment group and intraperitoneally injected with 0.1 mL of sterile saline (0.85%) containing 10^8^ CFU/mL virulent *A. hydrophila*. Fish were monitored twice daily, and individuals exhibiting severe stress, loss of equilibrium, or abnormal behavior were humanely euthanized using an overdose of MS‐222 and recorded as mortalities. Observations continued for 14 days to document cumulative mortality. To confirm the cause of death, samples were aseptically collected from moribund or freshly dead fish. *A. hydrophila* was reisolated and identified through biochemical and molecular methods, and the recovered strain was identical to the challenge strain, thereby fulfilling Koch’s postulates. The relative level of protection (RLP) was calculated following the formula described by Ruangpan et al. [39].

### 2.8. Statistical Analysis

All data are presented as mean ± standard error of the mean (SEM), with three replicates per treatment (*n* = 3). Differences among treatments were analyzed using one‐way analysis of variance (ANOVA), followed by Tukey’s multiple comparison test at a significance level of *p* < 0.05. Analysis of covariance (ANCOVA) was performed using CP, lipid, ash, and moisture as covariates to evaluate their influence on growth performance and immune responses. For data that did not meet normality assumptions, the nonparametric Kruskal–Wallis test was applied. All statistical analyses were performed using STATISTICA 12 software (StatSoft Inc., USA).

To further characterize the dose–response relationship and estimate the optimal dietary inclusion level of TML meal, a quadratic regression analysis was performed using the probiotic‐supplemented dietary treatments (0, 7.5%, 15%, and 30% TML inclusion). WG was used as the response variable, and the regression analysis was conducted in GraphPad Prism (version 11.0.2; GraphPad Software, San Diego, CA, USA). The quadratic model was fitted according to the equation: WG = *a* + *bX* + *cX* ^2^, where *X* represents dietary TML inclusion level (%), *a* is the intercept, *b* is the linear coefficient, and *c* is the quadratic coefficient. The optimum inclusion level was calculated from the fitted equation using *X*_opt_ = −*b*/2*c*. Model fit was evaluated using the coefficient of determination (*R* ^2^).

## 3. Results

### 3.1. Growth Performance, Body Proximate Composition, and FA Profiles

Dietary supplementation with yellow mealworm TML and probiotics resulted in significant increases in growth performance, feed utilization efficiency, and survival rates in Nile tilapia, as presented in Table 3. At the onset of the trial, all treatment groups presented statistically similar initial body weights (mean BW: 12.81 ± 1.37 g). However, by day 14, significant improvements in growth were observed (*p* < 0.05), with the T50P^+^ group (50% TML + 2% probiotics) showing a substantially greater mean BW (21.19 ± 1.18 g) than the CTRL group (20.04 ± 1.42 g). These differences persisted throughout the study, with the T50P^+^ group reaching a final BW of 78.62 ± 0.28 g (*p* < 0.05). The T100P and T50P^–^ groups also presented favorable results, with final BWs of 73.12 ± 1.69 g and 71.01 ± 1.14 g, respectively. However, both of these values were significantly lower than those in the T50P^+^ group, indicating that partial replacement of FM with TML in combination with probiotics was the most effective strategy for promoting growth in Nile tilapia. Throughout the experimental period, the CTRL group consistently presented the poorest performance, with the lowest WG and the lowest final body weight (FBW; 62.27 ± 1.84 g), which was significantly lower (*p* < 0.05) than those of all the other treatment groups. Similarly, the T50P^+^ group presented the highest survival rate (98.66 ± 0.11%, *p* < 0.05), which significantly surpassed that of the control (91.33 ± 3.7%). The WG and SGR followed the same trend, with T50P^+^ resulting in the highest WG (66.24 ± 1.36 g) and SGR (3.29 ± 0.05%/day), both of which were significantly greater than those of the control (49.18 ± 0.7 g and 3.11 ± 0.01%/day, *p* < 0.05). The FCR was significantly improved in the T50P^+^ group, with the lowest FCR (1.31 ± 0.02) (*p* < 0.05).

**Table 3:** Growth performance parameters of Nile tilapia (*Oreochromis niloticus*) fed experimental diets over an 8‐week trial period.

Parameters	Units	CTRL	T25P^+^	T50P^+^	T100P^+^	T50P^–^	CTRLP^+^
SR	%	91.33 ± 3.7^c^	95.64 ± 2.26^b^	98.66 ± 0.11^a^	96.11 ± 3.28^ab^	94.24 ± 3.87^bc^	94.47 ± 2.26^bc^
IBW	(g/biomass)	13.09 ± 1.14	12.41 ± 1.19	12.38 ± 1.64	13.34 ± 1.27	12.31 ± 2.54	13.28 ± 1.49
FBW	(g/biomass)	62.27 ± 1.84^d^	69.34 ± 1.26^c^	78.62 ± 0.28^a^	73.12 ± 1.69^b^	71.01 ± 1.14^c^	69.74 ± 0.98^c^
WG	(g/biomass)	49.18 ± 0.7^d^	56.93 ± 0.70^c^	66.24 ± 1.36^a^	59.78 ± 0.42^b^	58.7 ± 0.4^b^	56.46 ± 0.51^c^
FCR	—	1.53 ± 0.4^a^	1.43 ± 0.05^b^	1.31 ± 0.02^d^	1.36 ± 0.01^c^	1.40 ± 0.01^bc^	1.42 ± 0.02^b^
SGR	%	3.11 ± 0.01^d^	3.21 ± 0.04^c^	3.29 ± 0.05^a^	3.26 ± 0.06^b^	3.24 ± 0.03^bc^	3.13 ± 0.02^d^

*Note*: CTRL, control diet (with no insect meal (*Tenebrio molitor*) or probiotics); T25P^+^, T50P^+^, T100P^+^ includes 2% probiotics and substitutes 25%, 50%, or 100% FM with TML; T50P^−^, inclusion of 50% TML without probiotics; CTRLP^+^, commercial feed with 2% probiotics but no TML, respectively. Superscript letters (a, b, c, d) indicate statistically significant differences among treatments (*p* < 0.05). The values are presented as means ± SEM (*n* = 3 tanks per treatment).

Abbreviations: FBW, final body weight; FCR, feed conversion ratio; IBW, initial body weight; SGR, specific growth rate; SR, survival rate; WG, weight gain.

**Figure 1:**
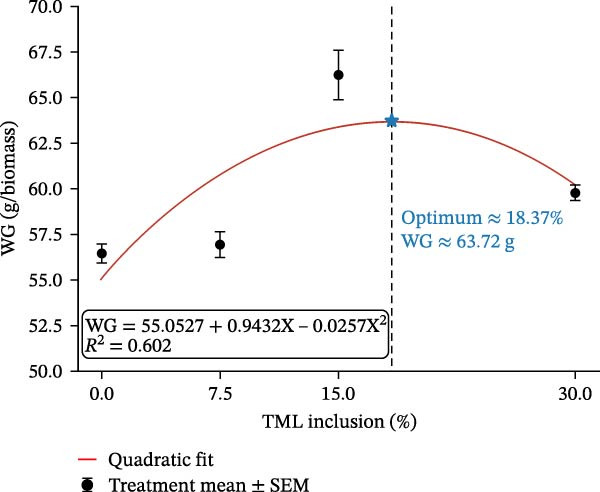
Quadratic regression of weight gain (WG) against dietary *T. molitor* meal (TML) inclusion level in Nile tilapia after 8 weeks of feeding. Points represent treatment means ± SEM. The fitted model was WG = 55.0527 + 0.9432X − 0.0257X^2^, where *X* is TML inclusion level (%). The predicted optimum TML inclusion level was 18.37%.

Quadratic regression analysis showed a curvilinear relationship between dietary TML inclusion level and WG (Figure 1). The fitted equation was WG = 55.0527 + 0.9432X − 0.0257X^2^, with *R* ^2^ = 0.602, indicating that 60.2% of the variation in WG was explained by TML inclusion level. The negative quadratic coefficient showed that WG increased up to a moderate TML level and then declined at higher inclusion. The predicted optimum TML inclusion was 18.37%, corresponding to an estimated WG of 63.72 g/biomass. This predicted optimum was close to the observed best treatment, T50P^+^, which contained 15% TML. These results suggest that ~15%–20% dietary TML inclusion is suitable for improving growth performance under the present experimental conditions.

In terms of body proximate composition, significant differences were observed among the experimental groups (Table 4). Compared with the CTRL group (23.46 ± 0.07% dry matter [DM]), the T50P^+^ group presented a significantly greater DM content (28.84 ± 0.01%, *p* < 0.05), likely due to a greater concentration of nutrients resulting from reduced water retention. Notably, the T50P^+^ group presented increased levels of crude lipids (CLs; 29.73 ± 0.51%, *p* < 0.05), CP (59.63 ± 0.26%, *p* < 0.043), and ash (17.98 ± 0.17%, *p* < 0.05) compared with those of the CTRL group (CL: 23.8 ± 0.48%, CP: 51.38 ± 0.29%, ash: 15.69 ± 0.060%). These findings indicate that the dietary inclusion of TML and probiotics enhances nutrient absorption and utilization. ANCOVA confirmed that the effects of dietary treatments on growth and immune performance remained significant after adjusting for variations in proximate diet composition (*p* < 0.05).

**Table 4:** Effects of dietary *Tenebrio molitor* (TML) meal and probiotics on the proximate composition and fatty acid profile of Nile tilapia (*Oreochromis niloticus*) after an 8‐week feeding trial.

Fatty acids	CTRL	T25P^+^	T50P^+^	T100P^+^	T50P^–^	CTRLP^+^
Proximate composition (%)						
CP	51.38 ± 0.29^d^	56.36 ± 1.73^b^	59.63 ± 0.26^a^	55.15 ± 0.93^b^	53.84 ± 0.03^c^	53.49 ± 0.11^c^
CL	23.8 ± 0.48^d^	26.57 ± 0.37^c^	29.73 ± 0.51^a^	30.58 ± 0.48^a^	28.47 ± 0.37^b^	26.12 ± 0.6^c^
Ash	15.69 ± 0.06^d^	17.59 ± 0.10^c^	17.98 ± 0.17^b^	17.67 ± 0.01^b^	17.83 ± 0.13^b^	16.79 ± 0.24^c^
DM	23.46 ± 0.07^d^	27.62 ± 0.9^b^	28.84 ± 1.01^a^	28.38 ± 0.55^a^	25.48 ± 0.12^c^	25.38 ± 0.17^c^
Fatty acid profiles (%)						
C14:0	4.49 ± 0.17^a^	4.35 ± 0.10^a^	3.93 ± 0.08^b^	3.71 ± 0.09^b^	3.64 ± 0.07^b^	3.52 ± 0.09^b^
C16:0	28.52 ± 0.76^a^	28.00 ± 0.30^ab^	29.10 ± 0.0^a^	28.489 ± 0.35^a^	28.59 ± 0.38^a^	27.53 ± 0.42^b^
C18:0	9.49 ± 0.68^a^	9.30 ± 0.10^a^	9.79 ± 0.15^a^	8.92 ± 0.12^b^	8.57 ± 0.14^b^	8.23 ± 0.14^b^
C18:1 n‐9	34.19 ± 0.31^b^	34.48 ± 0.42^b^	36.23 ± 0.45^a^	35.91 ± 0.50^a^	35.73 ± 0.40^a^	35.17 ± 0.45^a^
C18:2 n‐6	11.49 ± 0.42^a^	11.16 ± 0.35^a^	11.07 ± 0.50^a^	10.50 ± 0.32^b^	10.20 ± 0.40^b^	10.10 ± 0.35^b^
C18:3 n‐3	1.69 ± 0.05^b^	1.76 ± 0.08^a^	2.03 ± 0.06^b^	2.13 ± 0.07^a^	2.09 ± 0.06^a^	2.03 ± 0.05^a^
C20:4 n‐6 (ARA)	1.15 ± 0.09^a^	1.00 ± 0.05^ab^	1.19 ± 0.07^a^	0.97 ± 0.06^b^	0.93 ± 0.04^b^	0.93 ± 0.03^b^
C20:5 n‐3 (EPA)	1.61 ± 0.08^ab^	1.62 ± 0.11^ab^	1.66 ± 0.07^a^	1.53 ± 0.08^b^	1.43 ± 0.07^c^	1.34 ± 0.06^c^
C22:6 n‐3 (DHA)	3.76 ± 0.20^b^	4.18 ± 0.30^a^	4.61 ± 0.25^a^	4.39 ± 0.28^a^	4.20 ± 0.22^a^	4.10 ± 0.20^a^

*Note:* CTRL, control diet (with no insect meal (*Tenebrio molitor*) or probiotics); T25P^+^, T50P^+^, T100P^+^ includes 2% probiotics and substitutes 25%, 50%, or 100% FM with TML; T50P^−^, inclusion of 50% TML without probiotics; CTRLP^+^, commercial feed with 2% probiotics but no TML, respectively. ANCOVA was conducted to assess the effects of dietary CP, CL, ash, and DM on performance and immune variables. The treatment effects remained significant (*p* < 0.05) after adjustment. Different superscript letters (a–c) within the same row indicate significant differences among dietary treatments (Tukey’s multiple comparison test, *p* < 0.05). Values sharing the same superscript letter are not significantly different.

Abbreviations: ARA, arachidonic acid; CL, crude lipid; CP, crude protein; DHA, docosahexaenoic acid; DM, dry matter; EPA, eicosapentaenoic acid.

Concerning the FA profile, the results revealed substantial variations among the experimental groups (Table 4). The total saturated fatty acid (SFA) content increased progressively with higher levels of TML inclusion, rising from 43.72 ± 1.15% in the CTRL group to 49.55 ± 0.38% in T50P^+^ and 51.02 ± 0.84% in T100P^+^. However, this overall increase was not uniformly reflected across individual FAs as components such as myristic acid (C14:0), palmitic acid (C16:0), and stearic acid (C18:0) exhibited variable responses among dietary treatments.

Dietary interventions exerted a relatively modest effect on polyunsaturated fatty acids (PUFAs), particularly the n‐3 fraction. The T50P^+^ group showed a slight increase in total PUFAs (9.67 ± 0.85%) compared to the CTRL group (8.53 ± 1.02%). Notably, this group maintained or slightly enhanced levels of key long‐chain n‐3 FAs, including eicosapentaenoic acid (EPA; 1.66 ± 0.07%) and docosahexaenoic acid (DHA; 4.61 ± 0.25%). In contrast, both lower (T25P^+^) and higher (T100P^+^) replacement levels resulted in inconsistent or reduced concentrations of certain FAs.

**Figure 2:**
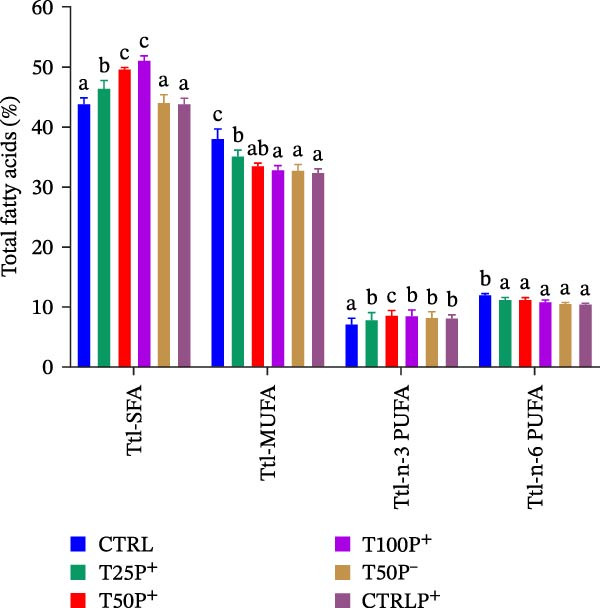
Total fatty acid composition (%) of Nile tilapia (*O. niloticus*) fed varying concentrations of *Tenebrio molitor* (TML) or probiotics over an 8‐week period. CTRL: control (no TML/probiotics); T25P^+^, T50P^+^, and T100P^+^: 25%, 50%, and 100% FM replaced with TML + 2% probiotics; T50P^–^: 50% TML without probiotics; CTRLP^+^: commercial diet + 2% probiotics. The values are the means (SEM). Different letters (a, b, c, d) indicate significant differences among groups (*p* < 0.05).

Monounsaturated fatty acids (MUFAs) declined in TML‐based diets relative to the CTRL group, with the most pronounced reduction observed in T50P^+^. Nevertheless, the inclusion of TML and probiotics was associated with an increase in oleic acid (C18:1n‐9) and α‐linolenic acid (ALA; C18:3n‐3), alongside a decrease in linoleic acid (C18:2n‐6). Consequently, the n‐3/n‐9 ratio decreased across treatments, from 2.03 ± 0.06 in the CTRL group to 1.69 ± 0.05 in T50P^+^, indicating a shift in FA profile and overall lipid balance (Figure 2).

### 3.2. Hematological Indices

**Figure 3:**
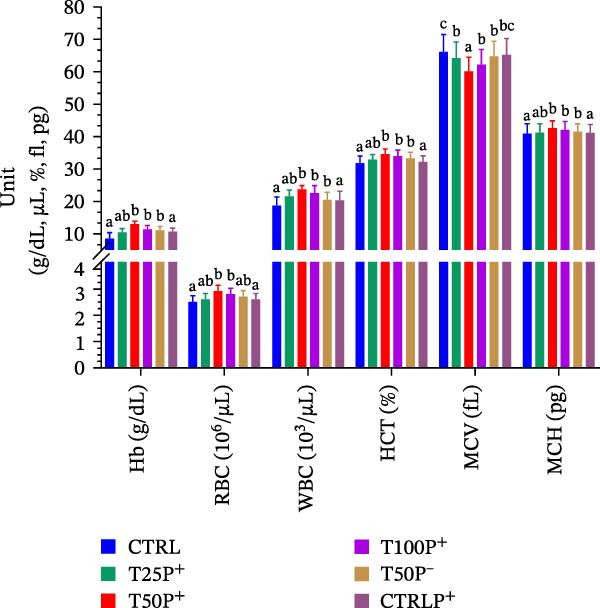
Hematological parameters of Nile tilapia (*O. niloticus*) fed varying concentrations of *Tenebrio molitor* (TML) or probiotics over an 8‐week period. The parameters included hemoglobin (Hb), hematocrit (HCT), red blood cell (RBC), white blood cell (WBC), mean corpuscular volume (MCV), and mean corpuscular hemoglobin (MCH) levels. CTRL: control (no TML/probiotics); T25P^+^, T50P^+^, and T100P^+^: 25%, 50%, and 100% FM replaced with TML + 2% probiotics; T50P^–^: 50% TML without probiotics; CTRLP^+^: commercial diet + 2% probiotics. The values are expressed as the means (SEM). Different letters (a, b, c, d) indicate significant differences among groups (*p* < 0.05).

The hematological profiles, including hemoglobin (Hb), RBC count, WBC count, HCT, MCV, and MCH, are depicted in Figure 3. The results revealed that the hemoglobin levels in the T50P^+^ (12.90 ± 0.9 g/dL) and T100P^+^ (11.18 ± 1.3 g/dL) groups were significantly greater than those in the CTRL group (8.50 ± 1.8 g/dL, *p*  < 0.05). Moreover, the elevated HCT values observed in the T50P^+^ and T100P^+^ treatments indicate an improved oxygen‐carrying capacity in fish fed diets supplemented with TML meal and probiotics, suggesting enhanced overall physiological conditions.

Similarly, the T50P^+^ group presented statistically significant increases in both the MCV (60.08 ± 4.5) and MCH (42.50 ± 2.5) (*p* < 0.05) relative to those of the other treatment groups, suggesting that the red blood cells in this group were both average in size and quantity. A similar trend was observed for the red blood cell (RBC) count, with significantly greater values in the T25P^+^ (2.60 ± 0.2 × 10^6^/µL) and T50P^+^ (2.90 ± 0.6 × 10^6^/µL) groups than in the CTRL (2.50 ± 0.2 × 10^6^/µL) group, suggesting an improvement in erythropoiesis. Additionally, both T25P^+^ and T50P^+^ fish groups presented increased white blood cell (WBC) counts (*p* < 0.05), indicating a strengthened immune response in these groups. These findings demonstrate that replacing FM with TML and probiotics, particularly in the T50P^+^ group compared with the T25P^+^ and T100P^+^ groups, had a significant positive effect on hematological health, leading to improved blood parameters and enhanced immune functions.

### 3.3. Lysozyme Activity

**Figure 4:**
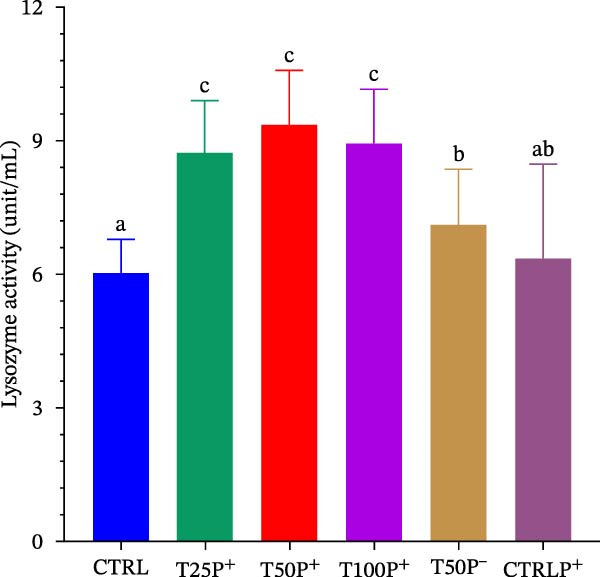
Serum lysozyme activity in Nile tilapia (*O. niloticus*) fed varying concentrations of *Tenebrio molitor* (TML) or probiotics over an 8‐week period. CTRL: control (no TML/probiotics); T25P^+^, T50P^+^, and T100P^+^: 25%, 50%, and 100% FM replaced with TML + 2% probiotics; T50P^–^: 50% TML without probiotics; CTRLP^+^: commercial diet + 2% probiotics. The values are expressed as the means (SEM). Different letters (a, b, c, d) indicate significant differences among groups (*p* < 0.05).

The lysozyme activity of the CTRL group was 6.02 ± 0.75 U/mg, serving as the reference point for comparison. Among the treatment groups, the T50P^+^ group presented the highest lysozyme activity at 9.37 ± 1.21 U/mg (Figure 4), indicating a statistically significant increase in the innate immune response relative to that of the control. Similarly, T25P^+^ and T100P^+^ also increased lysozyme activity, with values of 8.72 ± 1.18 U/mg and 8.94 ± 1.25 U/mg, respectively. On the other hand, the T50P^–^ and CTRLP^+^ groups presented lower lysozyme levels, at 7.13 ± 1.23 U/mg and 6.34 ± 2.14 U/mg, respectively, values that were slightly greater than those of the CTRL group but notably lower than those of the T50P^+^ group. These results indicate that the combined dietary inclusion of TML and probiotics, particularly at the 50% TML level, yields the most pronounced increase in lysozyme‐mediated immune function in Nile tilapia.

### 3.4. Digestive Enzyme Activity

**Figure 5:**
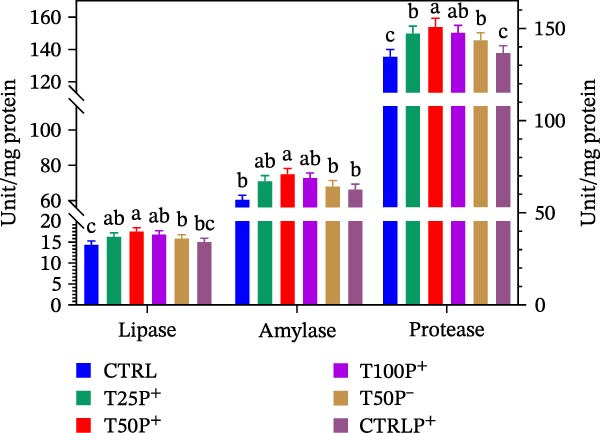
Digestive enzyme activities (amylase, protease, and lipase) in Nile tilapia (*O. niloticus*) fed varying concentrations of *Tenebrio molitor* (TML) or probiotics over an 8‐week period. CTRL: control (no TML/probiotics); T25P^+^, T50P^+^, and T100P^+^: 25%, 50%, and 100% FM replaced with TML + 2% probiotics; T50P^–^: 50% TML without probiotics; CTRLP^+^: commercial diet + 2% probiotics. The values are expressed as the means (SEM). Different letters (a, b, c, d) indicate significant differences among treatments (*p* < 0.05).

The substitution of the diet significantly influenced the activities of digestive enzymes, including lipase, amylase, and protease (Figure 5). To assess whether differences in proximate diet composition influenced growth and immune responses, ANCOVA was conducted with CP, CL, DM, and ash as covariates. The analysis revealed that the effects of dietary treatment on FBW, the FCR, lysozyme activity, and antioxidant enzyme levels (SOD, CAT) remained statistically significant (*p* < 0.05) after adjustment. These findings indicate that the observed biological improvements were not merely attributable to variations in nutrient composition but were significantly driven by the combined dietary effects of TML and probiotics.

The T50P^+^ group presented the highest amylase activity (72.67 ± 2.59 U/mg protein, *p* < 0.05), compared with the CTRL group (67.35 ± 2.87 U/mg protein). The T25P^+^ (71.02 ± 2.27 U/mg protein) and T100P^+^ (71.57 ± 3.05 U/mg protein) groups presented intermediate levels of amylase activity, with no significant difference observed between them and the T50P^+^ group. On the other hand, there were no notable differences between the T50P^–^ (69.21 ± 3.14 U/mg protein), CTRLP^+^ (68.28 ± 2.98 U/mg protein), and CTRL groups.

In terms of protease activity, T50P^+^ presented the highest level (151.85 ± 5.25 U/mg protein). Both T25P^+^ (146.67 ± 5.11 U/mg protein) and T100P^+^ (148.12 ± 4.99 U/mg protein) resulted in statistically significant increases in protease activity, although their levels were still lower than those observed in T50P^+^. In contrast, T50P^–^ (143.39 ± 4.95 U/mg protein) and CTRLP^+^ (135.45 ± 4.78 U/mg protein) did not result in notable increases. A similar trend was observed for lipase activity, with the T50P^+^ group exhibiting the highest activity (16.39 ± 0.81 U/mg protein, *p* < 0.05), which significantly surpassed that of the CTRL group (13.51 ± 0.33 U/mg protein). Additionally, the T25P^+^, T100P^+^, and T50P^-^ groups also presented elevated lipase activity compared with the control.

### 3.5. Antioxidant Status

**Figure 6:**
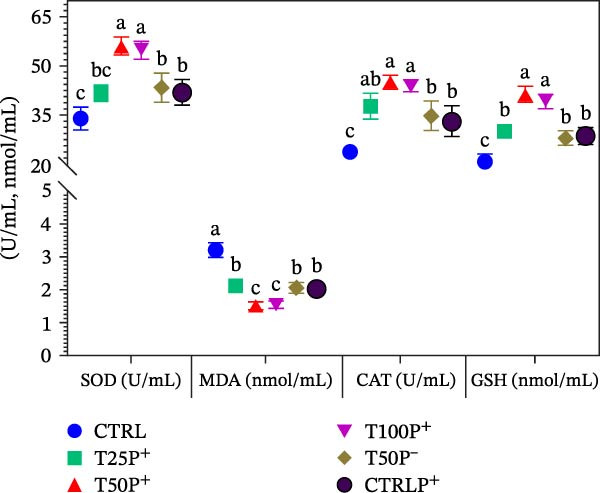
Antioxidant enzyme activities in Nile tilapia (*O. niloticus*) fed varying concentrations of *Tenebrio molitor* (TML) or probiotics over an 8‐week period. The parameters included superoxide dismutase (SOD), malondialdehyde (MDA), catalase (CAT), and glutathione peroxidase (GSH). CTRL: control (no TML/probiotics); T25P^+^, T50P^+^, and T100P^+^: 25%, 50%, and 100% FM replaced with TML + 2% probiotics; T50P^–^: 50% TML without probiotics; CTRLP^+^: commercial diet + 2% probiotics. The values are presented as the means (SEM). Different letters (a, b, c, d) indicate statistically significant differences among groups (*p* < 0.05).

Compared with those in the CTRL group, the antioxidant enzyme activities in all experimental groups were significantly greater (Figure 6). Notably, CAT activity was markedly elevated in the T50P^+^ and T100P^+^ groups. Additionally, the levels of SOD and glutathione significantly increased in the T25P^+^, T50P^+^, and T100P^+^ treatments, indicating a strengthened antioxidant defense system in response to dietary supplementation with TML meal and probiotics. The T50P^+^ group presented the highest antioxidant enzyme activities among all the treatments, with CAT at 46.21 ± 2.05 U/mL, SOD at 57.23 ± 2.71 U/mL, and GSH at 42.19 ± 2.42 nmol/mL, which were significantly greater than the corresponding values in the CTRL group (24.89 ± 1.55 U/mL for CAT, 35.10 ± 3.45 U/mL for SOD, and 23.14 ± 2.07 nmol/mL for GSH; *p*  < 0.05). Furthermore, both the T50P^+^ and T100P^+^ groups presented significantly reduced levels of MDA, indicating decreased lipid peroxidation and oxidative stress. The T50P^+^ group presented the lowest MDA concentration, at 1.49 ± 0.22 nmol/mL, compared with 3.22 ± 0.19 nmol/mL in the CTRL group (*p* < 0.05). Collectively, these findings indicate that the T50P^+^ diet provides the most effective antioxidant protection, enhancing the ability of fish to mitigate oxidative damage.

### 3.6. Bactericidal Activity

**Figure 7:**
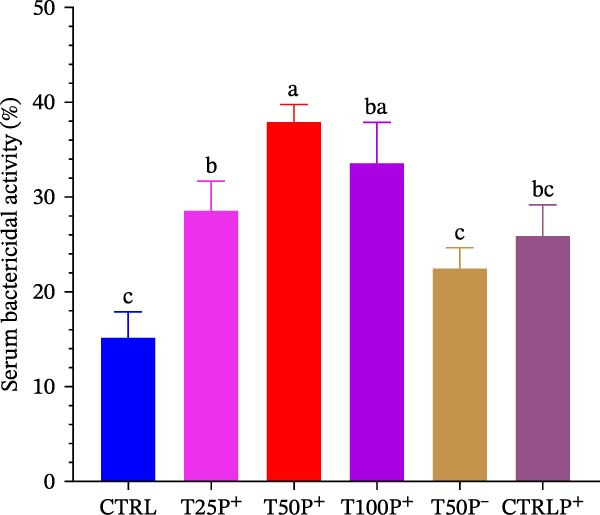
Prechallenge serum bactericidal activity in Nile tilapia (*O. niloticus*) fed varying levels of *Tenebrio molitor* (TML) and probiotics over an 8‐week period. CTRL: control (no TML/probiotics); T25P^+^, T50P^+^, and T100P^+^: 25%, 50%, and 100% FM replaced with TML + 2% probiotics; T50P^–^: 50% TML without probiotics; CTRLP^+^: commercial diet + 2% probiotics. The values are expressed as the means (SEM). Different letters (a, b, c, d) indicate statistically significant differences among groups (*p* < 0.05).

Serum bactericidal activity against *A. hydrophila* was significantly improved in fish fed diets supplemented with TML meal and probiotics (Figure 7). The T50P^+^ group presented the highest bactericidal activity at 38.23 ± 2.02%, which was significantly greater than that of the CTRL group (16.12 ± 0.37%; *p*  < 0.05). Similarly, the T100P^+^ group demonstrated a significantly greater bactericidal response (32.97 ± 1.19%) than did the CTRL group, although the response was slightly lower than that of the T50P^+^ group. These results indicate that while increased TML inclusion supports immune enhancement, the combined effect of 50% TML combined with probiotics (T50P^+^) yields the most robust stimulation of nonspecific immune defense in Nile tilapia. Additionally, the T25P^+^ and CTRLP^+^ groups presented significantly greater bactericidal activity (27.81 ± 1.27% and 25.16 ± 1.34%, respectively) than did the CTRL group (*p*  < 0.05). However, their activity levels remained lower than those observed in the T50P^+^ group. In contrast, the T50P^–^ group presented reduced bactericidal activity (23.09 ± 1.21%), which was significantly lower than that of the T50P^+^, T100P^+^, and T25P^+^ groups but still greater than that of the CTRL group. These results highlight the essential role of both TML meal and probiotics in enhancing the immune response of Nile tilapia, with the most pronounced immunostimulatory effect achieved through the combined inclusion of 50% TML and probiotics.

### 3.7. Experimental Challenge

**Figure 8:**
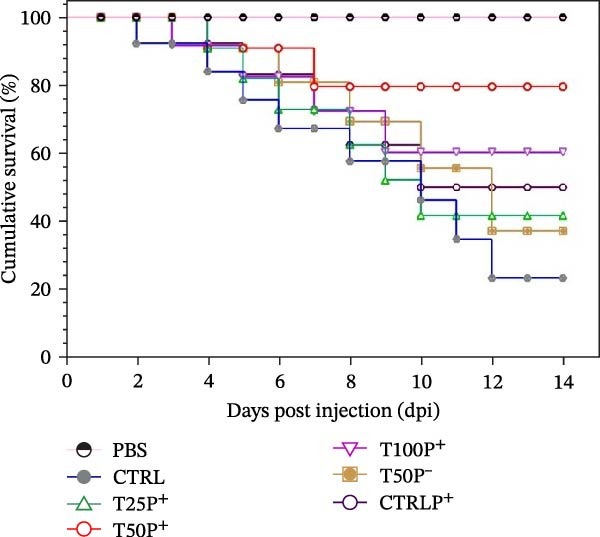
Cumulative survival (%) of *O. niloticus* over 14 days following intraperitoneal challenge with *A. hydrophila*. The fish were fed experimental diets for 8 weeks prior to infection. The treatments included the following: CTRL (control, no TML/probiotics), T25P^+^, T50P^+^, and T100P^+^ (25%, 50%, and 100% fishmeal replaced with *T. molitor* + 2% probiotics), T50P^-^ (50% TML without probiotics), and CTRLP^+^ (commercial diet with 2% probiotics). The PBS group was injected with sterile buffer and served as an unchallenged control.

Following *A. hydrophila* challenge, survival rates clearly differed across treatments (Figure 8). As expected, the PBS group presented the highest survival rate (100%). Among the challenged groups, T50P^+^ and T100P^+^ resulted in significantly greater survival than did the CTRL, with T50P^+^ maintaining ~ 80% survival on day 14. Moderate protection was observed in T25P^+^ and CTRLP^+^, both of which stabilized above 60%. In contrast, T50P^-^ and CTRL showed the steepest declines, with final survival rates falling below 40%. These findings highlight the combined role of TML and probiotics in enhancing resistance to *A. hydrophila*, with the 50% replacement level providing the most consistent protection. Notably, probiotics alone (CTRLP^+^) improved survival but not to the extent achieved by the combined TML–probiotic formulation. The successful reisolation of *A. hydrophila* from internal organs fulfilled Koch’s postulates, thereby confirming its causal involvement in the observed disease manifestations.

## 4. Discussion

The shortage of FM and rising costs have increased the demand for sustainable protein alternatives in aquaculture. However, plant‐based substances often have limitations due to antinutritional effects and nutritional imbalances. For example, soybean meal (SBM) can promote enteritis in Atlantic salmon, limiting nutritional intake and development [40], while pea‐ and wheat‐based proteins may lack essential AAs such as methionine and lysine in rainbow trout [41]. Rapeseed meal has also been associated with poor carp performance because it contains glucosinolates, which interfere with metabolism [42]. In contrast, insect meal has shown promising results as an aquafeed protein source, improving growth and feed efficiency in various aquatic species [43]. It also has environmental benefits as it reduces dependence on fish feed and promotes resource conservation [16]. Similarly, probiotics are appealing feed additives because of their availability, cost‐effectiveness, and capacity to enhance fish growth and immune responses when included in the diet [24, 44].

The study found that replacing FM with TML and probiotics (T50P^+^) dramatically enhanced Nile tilapia growth and survival rates. Fish in this group had a higher FBW (78.62 ± 0.28 g) and a higher survival rate (~98%) than the CTRL (*p* < 0.05). These findings are consistent with earlier research demonstrating that moderate inclusion of insect meal mixed with probiotics improves the development and feed efficiency [24, 45–47]. The better performance is most likely due to increased nutrient digestibility and metabolic efficiency. However, completely replacing FM (T100P^+^) resulted in lower performance compared to T50P^+^, despite surpassing the CTRL. This shows that excessive inclusion of insect meal may cause nutritional imbalance and decreased digestion. Quadratic regression analysis predicted an optimum dietary TML inclusion level of 18.37%, which closely agreed with the superior growth performance observed in the T50P^+^ group (15% TML). This finding confirms a dose‐dependent response and further supports that moderate TML inclusion is more effective than complete FM replacement under the present experimental conditions. Similar reductions in growth have been observed at high insect meal inclusion levels due to chitin content and amino acid restrictions [12, 44, 48]. These findings suggest that partial replacement is more effective than complete substitution.

Increased digestive enzyme activity supports improved growth performance. In this study, protease, lipase, and amylase activities were higher in the T50P^+^ group, indicating enhanced digestive capacity. Probiotics are known to stimulate endogenous enzyme secretion and improve gut microbial balance, which enhances nutrient digestion [49]. Additionally, probiotics may facilitate the breakdown of chitin present in insect meals, increasing nutrient availability. These combined effects likely contributed to improved feed utilization [50].

Dietary treatment, which also influenced the whole‐body composition. Compared with those in the CTRL group, the fish in the T50P+ group presented a greater CP content, which was likely attributable to the superior FA profile of TML and increased nutrient assimilation facilitated by probiotic supplementation. These findings are consistent with those of [24], who reported that the use of insect‐based proteins in conjunction with probiotics to replace FM partially increased protein levels in Nile tilapia (from 16.3 ± 0.1% to 16.7 ± 0.12%). The CL content was also significantly higher in the T50P^+^ group (29.73 ± 0.51%) than in the CTRL group (23.8 ± 0.48%), indicating greater lipid deposition and energy storage. Although ash and DM varied among treatments, statistical analysis confirmed that these differences did not account for the improved growth and immune responses. This indicates that the observed benefits are mainly due to the functional effects of TML and probiotics rather than basic nutrient composition [47, 51]. The DM content was also slightly but significantly elevated in the treated groups, which aligns with the findings of [47], who reported increased DM in fish fed insect‐based diets. Additionally, the T50P^+^ group presented a lower ash content (17.98 ± 0.17%), supporting previous observations by Mastoraki et al. [51] and indicating improved protein utilization and metabolic efficiency.

Although the proximate composition varied slightly among the experimental diets, ANCOVA confirmed that these differences did not account for the observed improvements in growth, feed efficiency, or immune performance. After adjusting for CP, lipid, and DM contents, dietary treatment remained a significant determinant of FBW, FCR, and innate immune responses. These results highlight the functional contributions of TML and probiotics beyond basic nutrition, underscoring their cooperative potential in tilapia diets.

The dietary supplementation had a clear effect on the FA profile of Nile tilapia; however, the response varied depending on the replacement dose. The current investigation found that TML inclusion led to an increase in total SFA (43.72% in CTRL to 49.55% in T50P^+^), which is comparable with previous findings that insect‐based diets increase saturated lipid fractions in fish tissues [52, 53]. However, this increase did not occur uniformly across all individual FAs, implying a shift in overall lipid distribution rather than simple accumulation. Moderate inclusion (T50P^+^) resulted in stable levels of essential FAs, with EPA and DHA reaching 1.66% and 4.61%, respectively, whereas excessive inclusion (T100P^+^) showed less consistent results.

Similar results were observed in tilapia and trout, where high insect meal reduced long‐chain n‐3 FAs due to their lower availability in insect lipids [54–56]. The decline in the n‐3/n‐6 ratio identified in this study adds to this trend and may affect fillet nutritional quality. These findings indicate that modest inclusion levels are better suited to maintaining a balanced FA composition. The T50P^+^ group had a superior lipid profile, possibly due to enhanced nutrition utilization from probiotic supplementation. However, no interaction impact was statistically assessed. The findings suggest that while TML can alter lipid composition, its presence should be carefully monitored to prevent harm to fillet quality.

Hematological profiling is a fundamental diagnostic tool for assessing fish health, offering critical insights into physiological status, stress responses, and immune competence. Erythrocytes (RBCs) are key indicators of systemic stress and play a central role in oxygen transport via hemoglobin (Hb), which directly influences tissue oxygenation [57]. Therefore, both the RBC count and hemoglobin concentration are essential parameters reflecting the efficiency of oxygen delivery and the metabolic capacity of a fish. In parallel, leukocytes (WBCs) are integral to innate immune defense, and their abundance serves as a reliable indicator of immunological activity and overall health status in fish [58]. The hematological results in this study further substantiate the immunostimulatory effects of the TML‐probiotic diet, with the T50P^+^ group demonstrating the highest RBC and WBC counts. These increases suggest enhanced oxygen‐carrying capacity and improved immune responsiveness. These findings are consistent with those of previous reports by Yonar et al. [59] and Wei et al. [60], which indicated that functional dietary supplements enhance hematological parameters and disease resistance in African catfish (*Clarias gariepinus*). Importantly, phagocytic cells, particularly neutrophils, are major contributors to circulating lysozyme levels, and elevated lysozyme activity has been closely associated with neutrophilia [61]. The TML‐probiotic combination in this study successfully supported nonspecific immune responses in Nile tilapia, as evidenced by the strong correlation between elevated serum lysozyme activity and the increase in WBC count observed in the T50P^+^ group.

In this context, a significant increase in lysozyme activity was observed in the T50P^+^ group, highlighting the potent immunostimulatory effects of TML combined with probiotics. The increase was significantly greater than that of the CTRL group, underscoring the role of these dietary additives in enhancing innate immune function. Lysozyme is a key component of the humoral arm of innate immunity, contributing to bactericidal defense by hydrolyzing the peptidoglycan layer of bacterial cell walls. Moreover, as an opsonin, lysozyme facilitates the activation of the complement system and promotes phagocytosis by enhancing the recognition and clearance of pathogens by immune cells [62]. These results correspond with those of [63], who reported a significant increase in lysozyme activity in tilapia fed insect‐based diets, with the activity increasing from 0.99 ± 0.04 U/mL in the CTRL group to 2.59 ± 0.12 U/mL in treated fish. Similar enhancements in immune parameters have been reported by Hoseinifar et al. [25] and Wang et al. [64], who reported that insect‐based diets supplemented with probiotics significantly increase innate immune responses across multiple fish species.

Moreover, the experimental diets substantially improved antioxidant activity, with the T50P+ group showing the highest levels of SOD, CAT, and GSH. These increased enzyme activities indicate enhanced oxidative stress regulation and cellular protection. These findings are consistent with previous studies suggesting that insect‐based meals, particularly when combined with probiotics, can enhance antioxidant defenses in fish [24]. In contrast, fish fed T50P^−^ presented reduced antioxidant enzyme activities, suggesting increased oxidative stress and diminished reactive oxygen species (ROS) scavenging capacity. The improved antioxidant response observed in the T50P+ group is likely attributable to the combined interaction between probiotics and TML, which may stimulate endogenous antioxidant enzyme production, as reported by Hoseinifar et al. [25].

Long‐term supplementation with TML and probiotics increased serum bactericidal activity against *A. hydrophila*, with the strongest response observed in the T50P^+^ group. This agrees with previous studies showing that insect‐based proteins and probiotics can enhance innate immunity and resistance to bacterial infection in fish [65]. The higher lysozyme activity observed in this group further supports improved antibacterial defense. In contrast, the T50P^-^ group showed lower bactericidal activity, indicating that TML alone was less effective than its combined use with probiotics. The improved immune response in the probiotic‐supplemented groups may be linked to the immunostimulatory effects of chitin and bioactive compounds in TML, as well as to the role of probiotics in modulating the gut microbiota and gut barrier function [66]. These findings support the use of balanced TML‐probiotic diets as a functional strategy to improve fish health and disease resistance.

The study found that Nile tilapia survival rates after *A. hydrophila* challenge differed significantly among dietary treatments, with the T50P^+^ group showing the most consistent protection (~80% survival). T100P^+^ also conferred high protection, while moderate benefits were observed for T25P^+^ and CTRLP^+^. In contrast, both the T50P^-^ and CTRL groups had poor survival (<40%). The present results are consistent with earlier studies demonstrating that probiotic supplementation, particularly with *Bacillus* spp., enhances disease resistance in tilapia challenged with *A. hydrophila* by improving survival, lysozyme activity, and antioxidant capacity [67]. Probiotics are known to potentiate innate immune responses through the stimulation of lysozyme, complement pathways, and antioxidant enzymes [68, 69].

Insect meals, particularly yellow mealworms, also contribute to improved health performance. Chitin and other bioactive compounds in insect‐derived feeds may act as immunostimulants or prebiotics, promoting beneficial effects on the gut microbiota and enhancing probiotic efficacy [70, 71]. This likely explains why TML combined with probiotics outperformed probiotics alone (CTRLP^+^). Indeed, the chitin‐rich exoskeleton of mealworms may provide a substrate for gut bacteria, enhancing the colonization and activity of beneficial strains [72, 73].

From a practical standpoint, partial replacement emerged as the most effective dietary strategy compared with complete substitution. This finding is highly relevant for aquaculture applications, as it indicates that TML can substantially reduce dependence on FM without incurring the performance limitations observed under full replacement. The improved FCR in the T50P^+^ group further reflects enhanced feed utilization efficiency, which may contribute to offsetting feed costs. Nevertheless, before formulating commercial recommendations, a comprehensive economic evaluation is warranted, encompassing ingredient pricing, feed processing costs, and the return on investment per unit of biomass gain.

## 5. Conclusion

In this study, partial FM replacement with TML, specifically at the 50% level combined with probiotics, consistently produced the greatest enhancements in growth performance, feed efficiency, innate immune responses, antioxidant status, and resistance to *Aeromonas hydrophila* in juvenile Nile tilapia. In contrast, complete FM replacement proved less effective, highlighting the importance of balanced inclusion. Nonetheless, further research is needed to refine formulation strategies and to assess the long‐term effects and economic feasibility under commercial aquaculture conditions.

## Author Contributions

Sivaramasamy Elayaraja, Channarong Rodkhum, and Vlastimil Stejskal conceived and designed the experiments. Sivaramasamy Elayaraja performed the experiments, analyzed the data, and drafted the manuscript. Sivaramasamy Elayaraja, Vlastimil Stejskal, and Channarong Rodkhum contributed to writing, reviewing, and editing the manuscript. Mahmoud Mabrok reviewed and edited the manuscript. Santosh Kumar Karn and Raja Subramaniyan edited and formatted the manuscript. Channarong Rodkhum and Vlastimil Stejskal were responsible for project administration, funding acquisition, conceptualization.

## Funding

This study was financially supported by the Second Century Fund (C2F) Chulalongkorn University, Thailand; the Ministry of Agriculture of the Czech Republic (NAZV Project QL24010346); and the Ministry of Education, Youth and Sports of the Czech Republic, OP JAK – project Aquaculture for Future (CZ.02.01.01/00/23_021/0012616).

## Disclosure

All the authors have read and agreed to the published version of the manuscript.

## Ethics Statement

Animal ethics. All animal handling and experimental protocols were carried out under the approval of the Institutional Animal Care and Use Committee (IACUC), Kasetsart University (Approval Number ACKU64‐FIS‐008), following the guidelines documented in the Ethical Principles and Guidelines for the Use of Animals for Scientific Purposes edited by the National Research Council of Thailand (NRCT).

## Conflicts of Interest

The authors declare that there are no conflicts of interest.

## Data Availability

The authors declare that they do not have any shared data available.
